# Risk of drug-induced angioedema: a pharmacovigilance study of FDA adverse event reporting system database

**DOI:** 10.3389/fphar.2024.1417596

**Published:** 2024-07-16

**Authors:** Maoxia Fan, Kaibin Niu, Xiaoqi Wu, Hongshuo Shi

**Affiliations:** ^1^ First Clinical Medical College, Shandong University of Traditional Chinese Medicine, Jinan, Shandong, China; ^2^ Department of Peripheral Vascular Surgery, Shuguang Hospital Affiliated to Shanghai University of Traditional Chinese Medicine, Shanghai, China

**Keywords:** angioedema, pharmacovigilance, fda, fares, adverse reaction, data mining

## Abstract

**Objective:**

The purpose of this study is to explore and analyze the FDA Adverse Event Reporting System (FAERS) database to identify drug adverse reaction signals associated with angioedema. The findings aim to provide valuable insights for clinical drug safety considerations.

**Methods:**

The Open Vigil 2.1 data platform was utilized to collect adverse event reports related to angioedema from the first quarter of 2004 to the fourth quarter of 2023. The reporting odds ratio (ROR) and proportional reporting ratio (PRR) were employed as disproportionality measures to detect adverse reaction signals Sof drugs associated with angioedema.

**Results:**

A total of 38,921 reports were retrieved, with the majority being reported by healthcare professionals. The analysis included predominantly adult patients (≥18 years of age), with slightly higher representation of females compared to males. Among the top 30 drugs associated with the occurrence of angioedema, 24 drugs showed positive signals in the risk analysis. Based on the individual drug reporting odds ratio (95% confidence interval) as a measure of risk signal strength, the top five drugs are as follows: lisinopril [ROR (95% CI): 46.43 (42.59–50.62)], enalapril [ROR (95% CI): 43.51 (39.88–47.46)], perindopril [ROR (95% CI): 31.17 (27.5–35.32)], alteplase [ROR (95% CI): 29.3 (26.95–31.85)], ramipril [ROR (95% CI): 20.93 (19.66–22.28)]. After categorizing the drugs, the strongest positive signal was observed in the antithrombotic agents [ROR (95% CI): 22.53 (21.16–23.99)], following that, cardiovascular drugs [ROR (95% CI): 9.17 (8.87–9.48)], antibiotics [ROR (95% CI): 6.42 (5.91–6.96)], immunosuppressors [ROR (95% CI): 5.95 (5.55–6.39)], anti-inflammatory analgesics [ROR (95% CI): 4.65 (4.45–4.86)], antiallergic drugs [ROR (95% CI): 4.47 (3.99–5)], antiasthmatics [ROR (95% CI): 2.49 (2.14–2.89)], blood sugar control drugs [ROR (95% CI): 1.65 (1.38–1.97)], and digestive system drugs [ROR (95% CI): 1.59 (1.45–1.74)] exhibited progressively decreasing ROR values.

**Conclusion:**

Many medications are associated with a high risk of angioedema. These medications play a crucial and potentially preventable role in controlling the occurrence of angioedema. It is essential to consider the risk level of drug-induced angioedema in clinical practice to optimize medication therapy.

## 1 Introduction

Angioedema is defined as a localized, non-inflammatory, self-limiting swelling caused by increased plasma leakage in the deep capillaries of the skin and mucous membranes (Hahn et al., 2017). The leakage relies on the accumulation of endogenous inflammatory compounds, which increase the permeability of endothelial cells without a complete inflammatory process. Therefore, among the four cardinal signs of inflammation described by Celsus (calor, dolor, tumor and rubor), only tumor (edema) is characteristic of angioedema (Cicardi et al., 2016; Kazandjieva and Christoff, 2019). Angioedema is often accompanied by other skin lesions, primarily rashes, which are caused by increased capillary leakage due to the action of endogenous mediators on the superficial vessels of the skin (Szymanski and Schaefer, 2023). Angioedema can occur with or without the presence of urticaria. It can be either inherited or acquired and is caused by various underlying mediators, including histamine and kinins (Sachs et al., 2018). The identification and understanding of the mediators responsible for inducing endothelial cell permeability and their release mechanisms are crucial factors in diagnosing and treating primary angioedema. The integrity of endothelial cell-cell junctions and the cell surface expression of transmembrane adhesion proteins and vascular endothelial (VE) cadherin play pivotal roles in vascular leakage and edema formation (Ashina et al., 2015). Furthermore, during episodes of angioedema, the detection of soluble VE-cadherin in patients’ serum further supports this notion, strengthening the aforementioned perspective (Bouillet and Vilgrain, 2014). The phosphorylation of intracellular proteins relies on the generation of nitric oxide (NO) by endothelial cell nitric oxide synthase (NOS). Various transmembrane receptors enhance endothelial cell NOS, thereby facilitating increased permeability. Two primary factors that determine vascular permeability are blood flow and endothelial barrier function (Curry and Adamson, 2010). Regarding histamine, recent research suggests that increased permeability is primarily due to NO-induced blood flow augmentation, regulated further by stimulation from vascular endothelial growth factor (Durán et al., 2010). Based on this discovery, wheals and flare reactions are characteristic features of histamine-mediated urticaria, with histamine-induced angioedema being more pronounced compared to non-histamine-induced angioedema (Cicardi and Zuraw, 2018).

In modern research, the classification of angioedema is based on its etiology, which can be categorized into three types: C1 inhibitor deficiency (C1-INH hereditary angioedema and C1-INH acquired angioedema), factor XII mutation (FXII hereditary angioedema), and angiotensin-converting enzyme inhibitor-induced angioedema (ACEI acquired angioedema) (Ashina et al., 2015; Cicardi et al., 2016). Angioedema frequently occurs in regions such as the face, neck, extremities, and mucosal linings area (such as the gastrointestinal tract) (Beavers et al., 2011; Bisinotto et al., 2019). When angioedema extends to the airways, it can rapidly become life-threatening, necessitating urgent intervention and emergency treatment (Gilbert and Byard, 2019). In recent years, there has been a remarkable advancement in our understanding of the fundamental biology of angioedema. Optimal treatment for patients with angioedema requires establishing an accurate diagnosis and implementing appropriate treatment based on the specific form of angioedema exhibited by each individual (Dubrall et al., 2020). The etiology of angioedema is complex and multifactorial, with medications being one of the significant risk factors that cannot be overlooked. Nonsteroidal anti-inflammatory drugs (NSAIDs), beta-lactam antibiotics, non-beta-lactam antibiotics, and angiotensin-converting enzyme inhibitors (ACEIs) are among the most common medications implicated in causing angioedema (Stone and Brown, 2017). Multiple studies have indicated that angioedema is a potentially life-threatening adverse reaction associated with ACEIs (Stone and Brown, 2017; Allihien et al., 2024; Mathey et al., 2024). A study assessed the association between medication therapy and the risk of angioedema, and the results indicated that taking multiple medications is considered a risk factor for the occurrence of angioedema due to adverse drug-disease or drug-drug interactions (Inomata, 2012).

As is widely known, the existing data primarily originates from clinical trials and observational studies, which inherently impose limitations on the populations, diseases, and medications involved. In the real world, there is currently a lack of more intuitive large-scale studies and relevant data regarding the adverse effects of angioedema. Therefore, our study utilized data from the US Food and Drug Administration (FDA) Adverse Event Reporting System (FAERS) to evaluate angioedema caused by the top 30 drugs. FAERS, the largest adverse event self-reporting database in the world, plays a crucial role in providing healthcare professionals and the public with post-marketing safety information regarding medications (Sakaeda et al., 2013). Despite the significant importance of identifying drugs that may lead to angioedema, no study to date has provided a comprehensive list of drugs associated with an increased risk of angioedema based on FAERS data. The objective of this study is to analyze adverse reactions of drugs reported in FAERS that are linked to angioedema and uncover potential risk signals associated with drugs that may increase the risk of angioedema. By identifying the risk levels that may contribute to an elevated risk of angioedema, this research aims to provide evidence for the selection of clinical medications and the reduction of angioedema occurrences, ultimately enhancing the safety of medication usage (Zhou et al., 2022).

## 2 Methods

### 2.1 Study design and data sources

The conducted study was a retrospective, observational pharmacovigilance investigation based on publicly available FAERS database (https://www.fda.gov/drugs/questions-and-answers-fdas-adverse-event-reporting-system-faers/fda-adverse-event-reporting-system-faers-public-dashboard). FAERS is a spontaneously maintained database managed by the FDA, collecting adverse events report information from various sources, including healthcare providers, patients, drug manufacturers, and other institutions (Sakaeda et al., 2013; Ahmad et al., 2022). The symptoms of AEs are coded using the Medical Dictionary for Regulatory Activities (MedDRA) (https://www.meddra.org/). MedDRA is an internationally standardized and clinically validated terminology system (Kumar, 2019). The Open Vigil 2.1-MedDRA tool (http://h2876314.stratoserver.net:8080/OV21d2/search/) was used to retrieve and extract relevant data.

The data collection period for this study spanned from 1 January 2004, to 31 December 2023, using the FAERS database. The inclusion criteria for this study were as follows: ① Adverse drug events (preferred term, PT: angioedema) occurring in patients. ② The scope of the study included adverse reaction reports of prescription drugs, biosimilars, and over-the-counter medications. ③ The collected data consisted of various components, including individual safety reports (ISR), demographic information-including patient’s age, gender, and reporter’s country (DEMO), adverse event records (REAC), drug utilization records (DRUG), report timestamps, and treatment outcome records.

### 2.2 Data processing procedure

In cases where the patient’s identification number, report date, and used medication are the same, duplicate entries are taken into consideration. One of the duplicate entries will be retained, while the others will be removed. If there are multiple records with the same patient ID for a particular medication, only the latest adverse event will be retained for statistical analysis. Each unique primary identifier in the dataset is assigned a single entry, and all preferred terms associated with adverse effects reported for that primary identifier are retained.

Medication standardized names can be referenced from databases such as Micromedex (https://www.micromedexsolutions.com/micromedex2/librarian) and Drug Bank (https://www.go.drugbank.com), which provide information on the generic names used in the United States. A formulation containing more than one active drug ingredient is defined as a combination product. Non-pharmaceutical products and drugs that are not marketed or have been withdrawn from the market in both China and the United States are excluded from consideration.

The categorization of medications is carried out by referencing the ACT codes (https://www.atcddd.fhi.no/atc_ddd_index/html), physiological systems and pharmacological mechanisms, with guidance from the UpToDate (https://www.sso.uptodate.com/contents/search) and Micromedex databases. This methodology enables the classification of drugs based on their therapeutic effects, physiological impact, and established classifications provided by the authoritative resources of UpToDate and Micromedex.

### 2.3 Statistical analysis

Adverse event signal detection was conducted using disproportionality analysis (DPA) with the reporting odds ratio (ROR) and the proportional reporting ratio (PRR) methods (Sakaeda et al., 2013). This method is based on a four-fold table (Table 1) and aims to identify potential adverse event signals by comparing the proportion of target events associated with the target drug to the proportion of target events associated with all other drugs. The ROR method utilizes a two-sided test with a 95% confidence interval (CI), where a lower limit greater than one indicates a signal, provided that the number of reports (a) is equal to or greater than 3. For the PRR method, the signal generation criteria include a minimum number of reports (a) of 3, a PRR value of two or higher, and a variance (χ2) of four or higher. The selected signals need to meet the criteria of at least one of the two methods, indicating a potential association between the drug and the event (Table 2). Count data is described using case numbers and proportions. All statistical analyses and visualizations were performed using R software (https://www.r-project.org/; version 4.0.0).

**TABLE 1:** Four-fold table of disproportionality methods.

Item	Number of target adverse event reports	Number of other adverse event reports	Total
Target drug	*a*	*b*	*a+b*
Other drugs	*c*	*d*	*c + d*
Total	*a+c*	*b + d*	*N = a+b + c + d*

**TABLE 2:** Formulas and threshold values of ROR and PRR.

Methods	Formula	Threshold value
ROR	$\text{ROR}=\frac{\left(a/c\right)}{\left(b/d\right)}=ad/bc$	*a*≥3; A signal is generated if the lower limit of 95%Cl of ROR>1
	$95\%\text{CI}=e^{\text{In}\left(\text{ROR}\right)\pm 1.96}\sqrt{\frac{1}{a}+\frac{1}{b}+\frac{1}{c}+\frac{1}{d}}$	
PRR	$\text{PRR}=\frac{a/\left(a+b\right)}{c/\left(c+d\right)}$	*a*≥3; PRR≥2, χ²≥4, a signal is generated
	$X^{2}=\frac{{\left(\left|ab-cd\right|-N/2\right)}^{2}\times N}{\left(a+b\right)\left(a+c\right)\left(c+d\right)\left(b+d\right)}$	

Note: ROR: reporting odds ratio, PRR: proportional reporting ratio.

## 3 Results

### 3.1 Descriptive analysis

#### 3.1.1 The process for retrieving AE reports for the target medication

**FIGURE 1:**
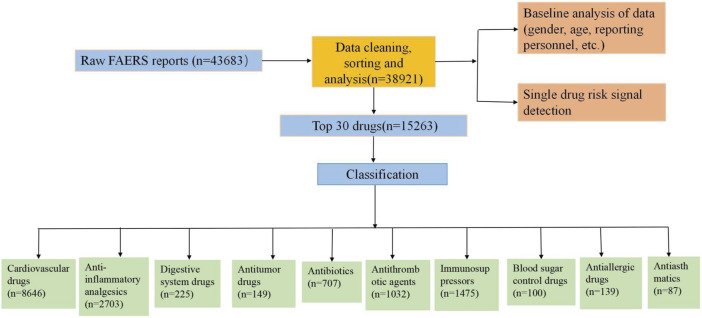
Flow chart for identification of angioedema reports of suspected.

A total of 43,683 reports were retrieved from the OpenVigil 2.1 database. After data cleaning, organization, and analysis, a total of 38,921 complete reports on angioedema were collected. Subsequently, a detailed analysis was performed on the top 30 ranked medications. For a more comprehensive understanding of the process, please refer to flowchart (Figure 1).

#### 3.1.2 Basic information of patients were included in the analysis

As shown in Table 3, a total of 38,921 patient cases were included in the analysis. Regarding gender composition, females (17,276 cases, 46.9%) were slightly higher than males (17,139 cases, 44.0%). This suggests that the risk of angioedema occurrence may not differ significantly between genders. In terms of age composition, the majority of cases were in adults (≥18 years old), with a total of 28,732 cases, accounting for 73.8% of the total cases.

**TABLE 3:** Clinical characteristics of reports with angioedema.

X	Overall (N = 38,921)	%
SEX		
Female	18,276	46.9
Male	17,139	44.0
Other	268	0.8
NA	3238	8.3
AGE(year)		
<18	1901	4.9
≥60	14,082	36.2
18–60	14,650	37.6
NA	8288	21.2
OCCP_COD		
Consumer	5394	13.8
Professional medical staff	28,427	72.8
Other	3518	9.3
NA	1,582	4.0

REPORTER_COUNTRY	The number of angioedema reports	%
The United States (US)	17,210	44.1
France (FR)	3143	8.0
The United Kingdom (UK)	3015	7.6
Canada (CA)	1977	5.0
Italy (IT)	1,656	4.2
Germany (DE)	1,287	3.3
Spain (ES)	1,236	3.1
Portugal (PT)	831	2.1

Report year	The number of angioedema reports reported
2004	687
2005	647
2006	718
2007	717
2008	870
2009	823
2010	1120
2011	646
2012	1184
2013	1376
2014	1511
2015	2348
2016	2291
2017	2398
2018	3341
2019	3137
2020	3121
2021	2004
2022	2634
2023	3087

Outcomes	The number of angioedema reports	%
Hospitalization (HO)	15,036	30.1
Congenital anomaly (CA)	25	0.1
Disability (DS)	643	1.2
Life-threatening (LT)	6,088	12.2
Death (DE)	724	1.4
Required intervention to prevent permanent impairment (RI)	2029	4.0
Other (OT)	22,650	45.4
NA	2,612	5.2

**FIGURE 2:**
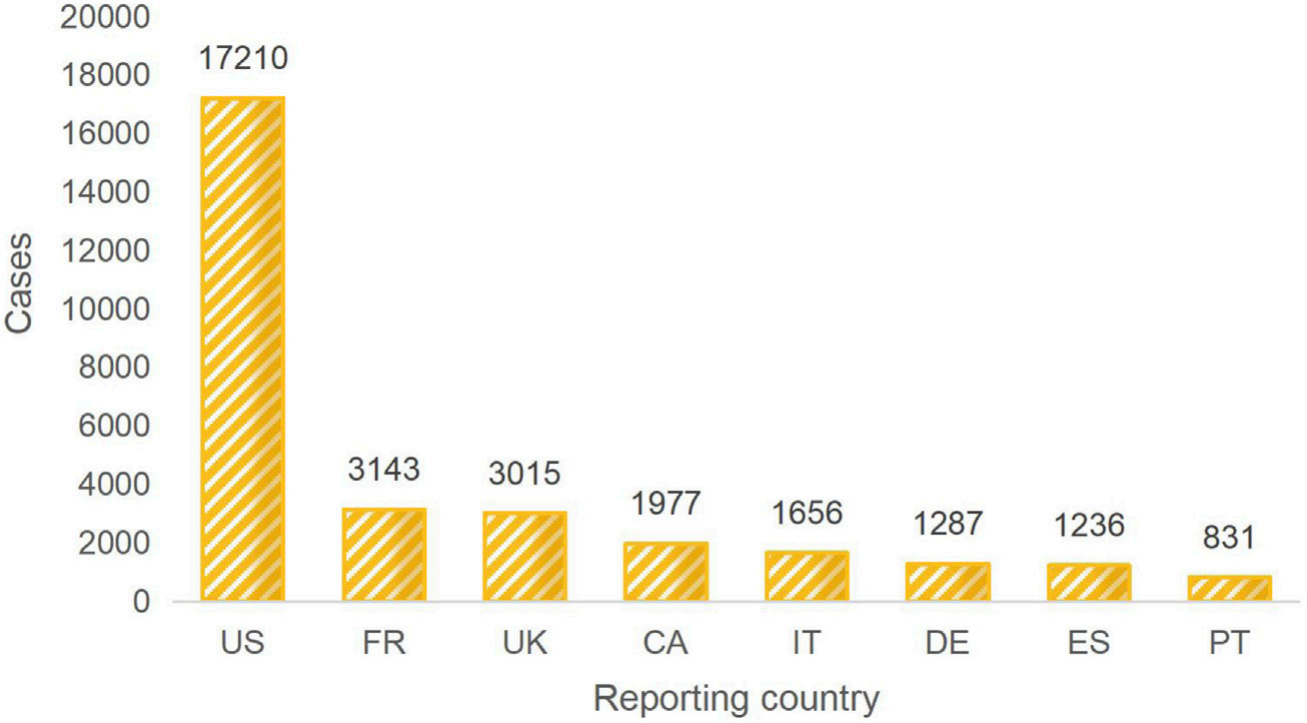
TOP 8 Reporter country. Note: US:The United States, FR: France, UK:The United Kingdom, CA:Canada, IT: Italy, DE:Germany, ES:Spain, PT:Portugal.

**FIGURE 3:**
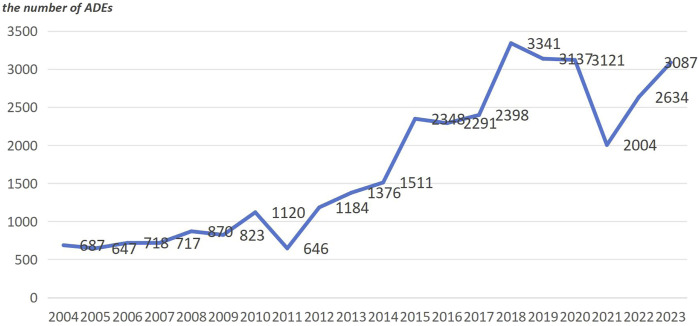
Distribution of angioedema related adverse drug events reporting year.

The majority of the reporters were professional healthcare workers (28,427 cases, 72.8%). This indicates a certain level of credibility for the reported adverse reactions. The data is derived from reports submitted by multiple countries. Among them, the United States ranks first in terms of the number of reports, followed by France, the United Kingdom, Canada, Italy, Germany, Spain, and Portugal. Collectively, these countries account for 77.4% of the reports. China contributed only 176 reports, while 1,432 reports did not specify the reporting country, representing 3.6% of the total. For a detailed distribution, please refer to Figure 2. The number of reports has shown fluctuating growth since 2004. In 2018, it reached its peak, and although there was a decline from then until 2021, the trend shows an increasing number of reports in 2022 and 2023. For a yearly breakdown of the number of reports, please refer to the line graph in Figure 3.

**FIGURE 4:**
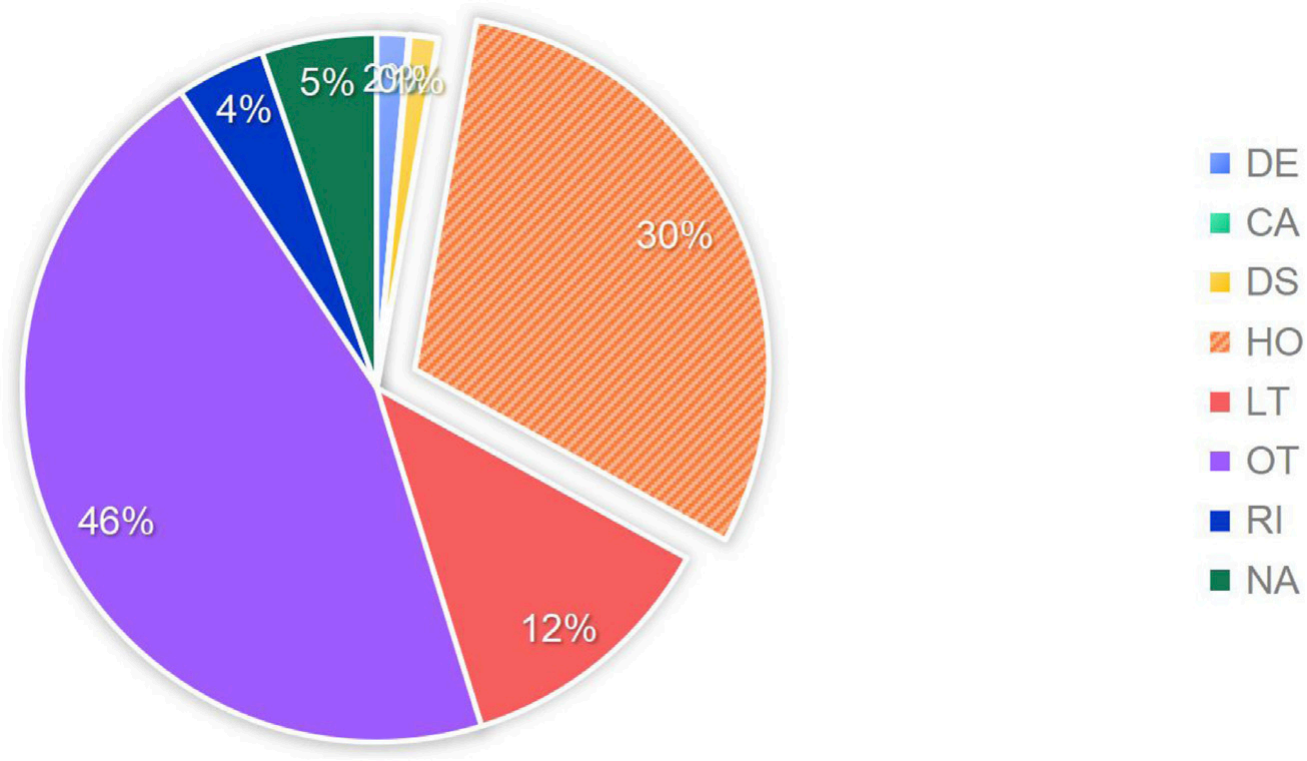
Proportion of adverse drug events included in the study outcomes. Note: DE:death, CA:congenital anomaly, DS:disability, HO:hospitalization, LT:life-threatening, OT:other, RI:required intervention to prevent permanent impairment, NA:not available.

According to the setup of the FAERS database, multiple outcomes can be selected. Therefore, for this study, the most severe outcome among the multiple choices was selected as the final outcome. The outcome with the highest proportion was “hospitalization or prolongation of hospitalization” with a total of 15,036 cases, accounting for 30.1%. The outcomes that posed a risk to patients and resulted in patient death totaled 6,812 cases, accounting for 13.6%. For a detailed distribution, please refer to Figure 4.

### 3.2 Drugs causing an increased risk of angioedema

To assess the risk signals associated with the various medications causing angioedema, the analysis focused on the top 30 drugs with representative reporting numbers. The top 30 drugs causing angioedema were classified into the following 10 categories: cardiovascular drugs, anti-inflammatory analgesics, antithrombotic agents, antibiotics, digestive system drugs, immunosuppressors, blood sugar control drugs, antiallergic drugs, antiasthmatics, antitumor drugs.

**FIGURE 5:**
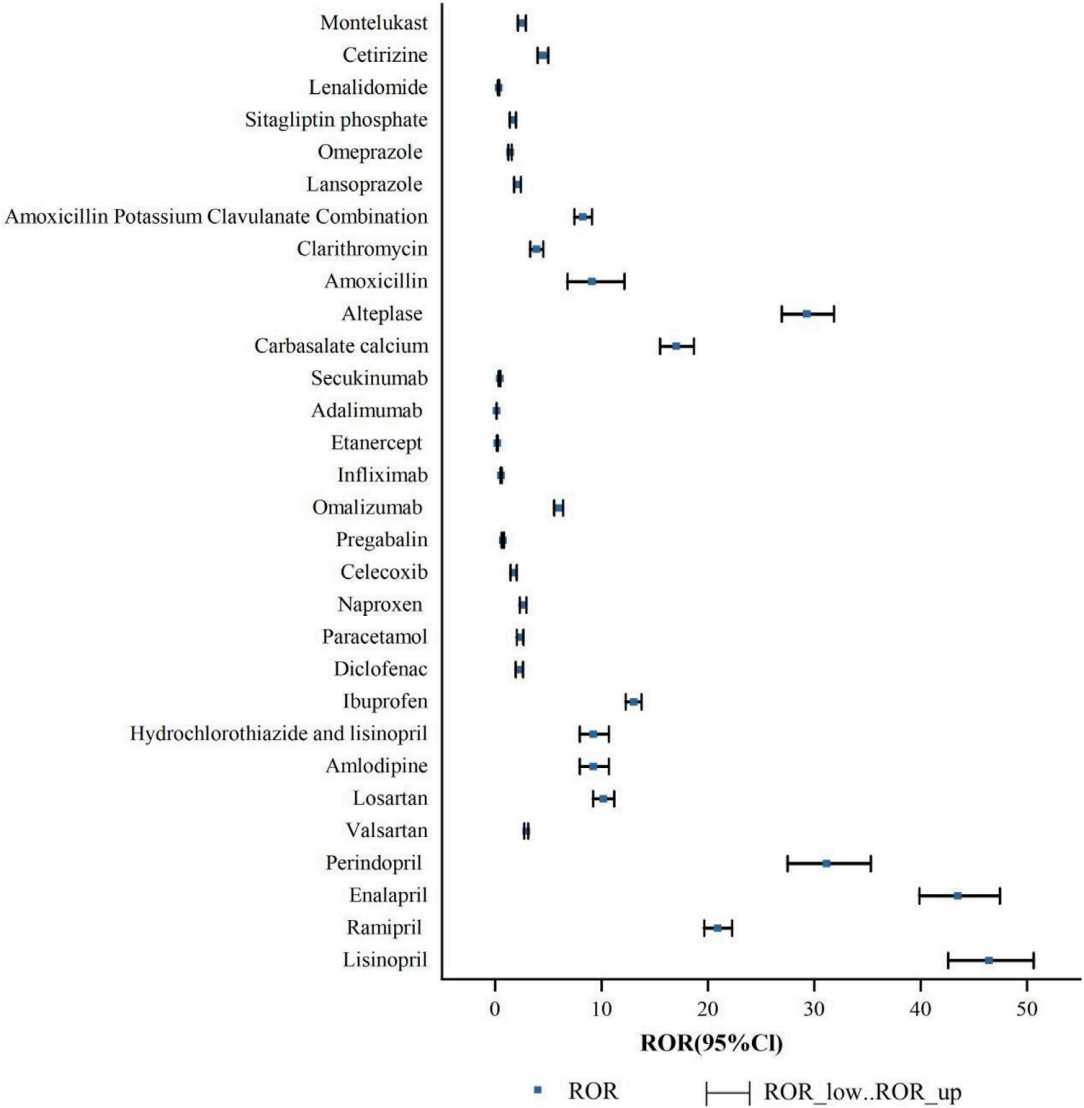
ROR for angioedema of single drug.

**FIGURE 6:**
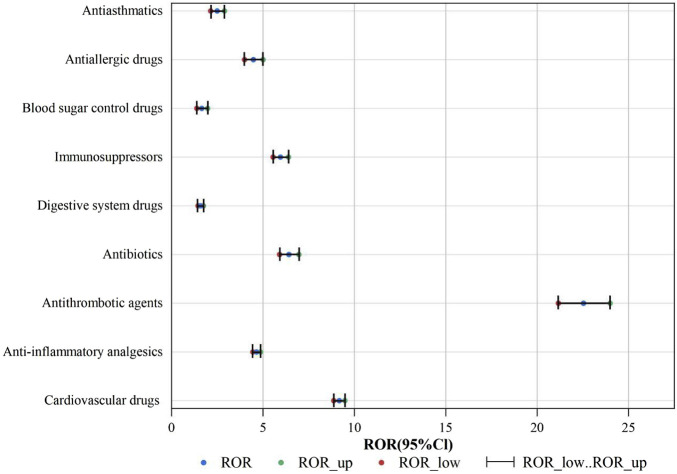
ROR for angioedema of each group of drugs.

An analysis was conducted to assess the strength of risk signals for individual drugs, as depicted in Figure 5. A comprehensive summary of the detailed analysis can be found in Supplementary Table S1. A total of 24 drugs were identified with positive signals for angioedema. These drugs were further categorized, and the strength of the risk signals was re-evaluated. Please refer to Figure 6 for specific details, and a comprehensive summary of the detailed analysis can be found in Supplementary Table S2.

#### 3.2.1 Single drug risk signal detection

Based on the ROR risk signal strength, the top five drugs with positive signals were as follows:lisinopril [ROR (95% CI): 46.43 (42.59–50.62)], enalapril [ROR (95% CI): 43.51 (39.88–47.46)], perindopril [ROR (95% CI): 31.17 (27.5–35.32)], alteplase [ROR (95% CI): 29.3 (26.95–31.85)], ramipril [ROR (95% CI): 20.93 (19.66–22.28)]. Other positive signal drugs are in order of risk signal intensity: carbasalate calcium, ibuprofen, losartan, amlodipine, hydrochlorothiazide and lisinopril, amoxicillin, amoxicillin potassium clavulanate combination, omalizumab, cetirizine, clarithromycin, valsartan, naproxen, montelukast, paracetamol, diclofenac, lansoprazole, celecoxib, sitagliptin phosphate, omeprazole. Indeed, a higher ROR indicates a stronger risk signal, suggesting an increased risk of angioedema. A larger ROR value signifies a higher likelihood of the adverse event being associated with the use of a specific drug. Therefore, drugs with higher ROR values are more strongly linked to the risk of angioedema.

The risk signal detection for angioedema associated with the drugs (pregabalin, infliximab, secukinumab, lenalidomide, etanercept and adalimumab) resulted in negative findings. These drugs did not show any significant signals indicating an increased risk of angioedema.

#### 3.2.2 Risk signals after classification of drugs

The positive signal groups were cardiovascular drugs [ROR (95%CI):9.17 (8.87–9.48)], anti-inflammatory analgesics [ROR (95%CI):4.65 (4.45–4.86)], antithrombotic agents [ROR (95%CI):22.53 (21.16–23.99)], antibiotics [ROR (95%CI):6.42 (5.91–6.96)], digestive system drugs [ROR (95%CI):1.59 (1.45–1.74)], immunosuppressors [ROR (95%CI):5.95 (5.55–6.39)], blood sugar control drugs [ROR (95%CI):1.65 (1.38–1.97)], antiallergic drugs [ROR (95%CI):4.47 (3.99–5.0)], antiasthmatics [ROR (95%CI):2.49 (2.14–2.89)]. The strongest risk signal was antithrombotic agents, followed by cardiovascular drugs, antibiotics, immunosuppressors, anti-inflammatory analgesics, antiallergic drugs, antiasthmatics, blood sugar control drugs, and digestive system drugs.

## 4 Discussion

This study presents a comprehensive and systematic analysis of adverse events related to angioedema in the FAERS database from 2004 onwards. Additionally, it explores the pharmacological risks associated with the development of angioedema. Angioedema primarily occurs in adults, and the data for this condition mainly originates from reports submitted by healthcare professionals. Therefore, the reliability of the data source is high. Angioedema can lead to hospitalization or prolongation of hospital stays and, in some cases, even death. These findings underscore the profound seriousness of angioedema as an adverse event. The association between angioedema adverse events and 24 drugs that have shown positive signals in causing angioedema was quantified using the ROR and PRR methods. These drugs were classified based on their physiological systems and pharmacological mechanisms. The positive signal drugs primarily belonged to the following categories: cardiovascular drugs, anti-inflammatory and analgesic drugs, antibiotics, immunosuppressants, antithrombotic drugs, gastrointestinal drugs, antiallergic drugs, antidiabetic drugs, and bronchodilators. Higher ROR values indicate a greater risk of angioedema occurrence associated with these drugs. This study represents the first exploration of the angioedema risk based on the FAERS database. It provides valuable evidence for reducing the incidence of angioedema and improving the rational use of medications. Moreover, it serves as a warning for healthcare professionals to take proactive measures when encountering angioedema cases and emphasizes the importance of enhancing medication safety surveillance during clinical practice to prevent the occurrence of angioedema events.

In recent years, as more drugs become available, the number of drugs that can induce angioedema has increased. Drug-induced angioedema has been reported to result from a wide range of drugs and vaccines, including NSAIDs, ACEIs, angiotensin II receptor antagonists, antibiotics, radiocontrast media, proton pump inhibitors, statins, fibrinolytic agents, estrogens, diuretics, calcium channel blockers, beta blockers, and psychotropic drugs (serotonin reuptake inhibitors) (Sánchez-Borges et al., 2002; Cicardi et al., 2004; Inomata, 2012). Drug-induced angioedema, similar to other cutaneous drug reactions, is most commonly reported to be caused by cephalosporin antibiotics and NSAIDs, although reliable data from epidemiological studies are scarce. In approximately 50% of cases, drug-induced angioedema is associated with urticaria and may potentially lead to life-threatening allergic reactions (Lerch, 2012). The symptoms of angioedema itself indicate that allergy is the most common cause, with histamine being the most frequent mediator. Once allergy is ruled out, and the symptoms recur, the determination of the underlying cause relies on identifying the mediators, which can be either histamine-mediated or non-histamine-mediated. Histamine accounts for nearly all cases of angioedema, with the majority of them lacking wheals (Cicardi and Zuraw, 2018). The first form of acquired angioedema, known as idiopathic histaminergic acquired angioedema, originates from an unknown source, although it ceases to recur after prolonged antihistamine therapy. The most common form of recurrent non-hereditary angioedema, even after high-dose antihistamine treatment, is associated with ACEIs (Kostis et al., 2005). ACEIs are widely used for the treatment of hypertension and provide cardiovascular and renal protection for patients with heart failure, chronic kidney disease, and those at high risk of cardiovascular events. The issue of adverse reactions related to ACEIs is clinically relevant due to the substantial number of individuals exposed to these medications, and this number is increasing (Weber and Messerli, 2008). Recent studies suggest that the risk of inducing angioedema with angiotensin receptor blockers (ARBs) after ACEIs-induced angioedema is likely no greater than with other antihypertensives. Given their cardioprotective properties, ARBs may be prescribed with caution after ACEIs-induced angioedema, especially in patients with cardiovascular risk factors (Rasmussen et al., 2019).

Approximately 0.5%–1% of treated patients are susceptible to recurrent angioedema, and the symptoms do not manifest immediately like drug adverse reactions. Instead, they may start years after initiating treatment, with varying frequencies of recurrence ranging from several times a year to weekly occurrences (Mansi et al., 2015). When ACEIs are discontinued, the swelling ceases or significantly decreases, although patients who experienced their first angioedema episode during treatment may continue to have recurrences after discontinuation (Carucci et al., 2020).

ACEIs have a greater impact on the oropharyngeal and perioral regions compared to other areas, and they may involve the throat, posing a risk of life-threatening complications (Beltrami et al., 2011). Bradykinin is believed to be involved in this form of angioedema, as ACE is the primary physiological pathway for bradykinin degradation (Wood et al., 1987). Evidence suggests that patients receiving ACEIs exhibit elevated plasma levels of bradykinin, particularly in those experiencing angioedema symptoms while being treated with these medications (Pellacani et al., 1994; Obtułowicz, 2016). Extensive clinical evaluations of commonly used inhibitors of the renin-angiotensin system provide reliable data on the incidence and clinical manifestations of angioedema caused by these drugs. Drug-induced angioedema can be triggered by various major pathophysiological mechanisms, including IgE-mediated allergic reactions, aspirin and other NSAID intolerances, such as those due to pharmacological inhibition of cyclooxygenase and bradykinin-related reactions. Recent reports have highlighted significant differences in the clinical presentation of ACEI-related angioedema compared to allergic reactions and NSAID intolerance.

Currently, there is insufficient data on the prevention and management of drug-induced angioedema. In contrast to drug-induced allergic angioedema, there are no established treatment methods specifically for drug-induced bradykinin-mediated angioedema. The trend of drug-induced angioedema can be changed as various new drugs enter the market. Therefore, it is important to monitor instances of angioedema occurring in association with any medication to gather crucial data and identify predisposing factors for drug-induced angioedema. This ongoing monitoring can help improve our understanding of this condition and enhance patient safety. Absolutely, sharing collected data on drug-induced angioedema with healthcare professionals in a timely manner is crucial for appropriate diagnosis and management of this condition. By providing healthcare providers with comprehensive information, they can make informed decisions regarding treatment options and strategies to mitigate the risk of drug-induced angioedema. Open communication between patients and doctors is vital for optimizing patient care and safety. Recording a patient’s medical history is indeed the first step in prevention. Generally, patients with a history of angioedema should be particularly cautious. For example, if feasible, ACEIs should be avoided in patients who have experienced idiopathic angioedema. Since predictive factors for ACEI-related angioedema have not been established, all patients using ACEIs should be aware of the possibility of this adverse reaction. In patients with a history of angioedema, caution should also be exercised when considering the use of fibrinolytics or ARBs. Due to insufficient data to draw specific preventive conclusions for certain types of angioedema, it is not recommended to switch to compounds within the same class in patients who have experienced angioedema. Therefore, further monitoring is necessary in such cases.

This study has several limitations that should be acknowledged: ① Proportional imbalance analysis, while a statistical method to determine the correlation between targeted drugs and adverse reactions, cannot establish a definitive causal relationship between targeted drugs and drug-related adverse reactions. It also cannot exclude other confounding factors, such as underreporting of adverse events or concomitant medication usage (Sakaeda et al., 2013). ② The data in the FAERS database are spontaneously and voluntarily reported, which may be influenced by recent research or media attention, potentially leading to certain biases (Montastruc et al., 2006). ③ The FAERS database does not provide corresponding assessment scales or narrative data to confirm whether patients truly meet the criteria for angioedema, and the study includes a lack of data on comorbidities and concomitant medications that could influence the occurrence or exacerbation of angioedema. ④ The FAERS database does not provide information on the incidence of adverse events in the general population. ⑤ Although the dataset included in this study is relatively large, it is advisable to incorporate data from other existing databases for further validation.

## 5 Conclusion

Angioedema is a commonly reported cause of drug adverse events in the FAERS database, indicating a widespread presence of medications associated with an increased risk of angioedema. It is advisable for clinical practice to consider the risk level of drug-induced angioedema and enhance medication safety monitoring during the application process. This proactive approach aims to prevent adverse events, such as angioedema, and optimize medication therapy.

## Data Availability

The original contributions presented in the study are included in the article/Supplementary Material, further inquiries can be directed to the corresponding authors.
